# A new clinical prognosis model for breast cancer with ADSS as the hub gene

**DOI:** 10.7150/jca.95589

**Published:** 2024-09-16

**Authors:** Zhi Zhang, Fei Xu, Suna Zeng, Xiaoying Li, Yuzhe Cai, Jinghua Li, Zha Peng, Yixuan Chen, Chengyu Huang, Ting Li, Steven Mo, Tongling Zhao, Hai Huang

**Affiliations:** 1 Department of Hepatobiliary Surgery, Guangxi Medical University Affliated Wuming Hospital, Nanning 530199, China.; 2 Experimental Center of BIOQGene, YuanDong International Academy of Life Sciences, Hong Kong 999077, China.; 3 School of Public Health, Southern Medical University, Guangzhou, China.; 4 Department of Stomatology, The First Affiliated Hospital of Guangxi Medical University, Nanning, China.; 5 The Graduate School of Guangxi Medical University, Nanning, China.; 6 Department of General Surgery, Guangxi Medical University Affliated Wuming Hospital, Nanning 530199, China.; 7 Systems Biology Research Center, Biology Institute, Guangxi Academy of Sciences, Nanning 530007, Guangxi, China.; 8 Departments of Developmental Planning, Guangxi Medical University, Nanning, China.

**Keywords:** ADSS, BRCA, WGCNA, clinical indicator model, prognosis

## Abstract

**Background:** Breast cancer (BRCA) is the most common malignant tumor and the leading cause of cancer death worldwide. Adenylosuccinate synthetase (ADSS) is highly expressed in BRCA and its subtypes malignant tumors and is associated with poor prognosis.

**Methods:** By applying ROC curve, survival analysis, WGCNA, enrichment analysis, Cox regression model and other methods, this study explores the role of ADSS in BRCA and constructs a scoring model.

**Results:** In this study, the ADSS demonstrated good diagnostic efficacy and high expression in breast cancer tissues. Further exploration of the role of ADSS in BRCA revealed that its significantly related coexpressed genes are clearly involved in biological functions and signaling pathways associated with cell proliferation and differentiation. Additionally, the ADSS-related scoring model showed a significant prognostic impact on clinical characteristics, such as metastasis to lymph nodes, and it was discovered that the ADSS score and related scoring genes may affect the immune microenvironment of BRCA patients, potentially participating in the occurrence of this disease.

**Conclusion:** In summary, our gene expression analysis of ADSS in BRCA generated a clinical scoring model based on the ADSS that may be used to assess prognostic risk and provide potential clinical applications and rational therapeutic targets.

## Introduction

Breast cancer (BRCA) is the most common malignancy worldwide and a major cause of death 1. Over the past 25 years, BRCA mortality has increased significantly worldwide 2, and an approach for treating BRCA is urgently needed, BRCA remains a major health problem 3. Currently, breast X-ray photography is the most commonly used method for BRCA detection. However, due to the large amount of data, early diagnosis of BRCA is very difficult 4. Mastectomy is considered the most effective method for treating advanced BRCA; however, tumor resection is not definitive. Unrealistic, deadly, and expensive treatments for BRCA are not helpful for many women worldwide who are at risk of BRCA 5.

Exploring tumor markers using bioinformatics techniques is an important approach. The discovery of BRCA stem cells revealed the pathogenesis and drug resistance mechanism of BRCA and revealed that many genes are involved in this disease 6. Adenosuccinate synthetase (ADSS) showed moderate cytoplasmic immunoreactivity in the majority of malignant cells, and high expression of ADSS was detected in a few cases of malignant lymphoma, breast cancer and colorectal cancer 7. Genetically, genetic variation or polymorphism governs gene expression and poses disease risk in humans 8, 9. The ADSS sequence is 44 kb in length and contains 13 exons and 12 introns. Many cancers require ADSS to synthesize nucleic acids, and by inhibiting adenosuccinate synthase, it is possible to find a new therapeutic agent 10. However, previous studies confirmed that ADSS was highly expressed in these diseases but did not further explore whether the dysregulated BRCA gene was associated with the overexpression of this gene. These dysregulated genes may also play important roles in this disease, which deserves further exploration.

In conclusion, in this study, we identified BRCA dysregulation genes associated with high ADSS expression and performed a weighted gene coexpression network analysis (WGCNA). Based on the module genes with the highest correlation with ADSS, we further developed a clinical model based on ADSS, revealing the multiomics landscape of the global regulatory network.

## Materials and Methods

### Organizational source

In this study, 30 breast samples were collected from Wuming Hospital affiliated to Guangxi Medical University, including 15 normal breast tissues and 15 BRCA tissues. Our inclusion criteria are strictly limited to adult female patients who are first diagnosed with invasive BRAC and have been followed up for more than 12 months. All samples were diagnosed as invasive cancer before surgery and collected after mastectomy. We excluded patients under the age of 18, patients undergoing prophylactic mastectomy, patients undergoing breast conserving therapy, and patients with rare tumor types such as lobular sarcoma and lymphoma. Additionally, for the collection and preservation of breast tissue, we have adopted a meticulous operational procedure. During the sampling and fixation process, it is necessary to use sharp instruments, preferably thin surgical blades, and minimize the use of scissors to avoid crushing and contusion of the BRCA specimens. Accurate acquisition of BRCA tissue is required, with specimens being as far away from the BRCA tissue area as possible; efforts should be made to ensure that the samples are placed in an appropriate 4% paraformaldehyde solution and fixed at room temperature. The volume of the fixing solution should be 10 times that of the tissue volume, and the optimal fixation time is 6-24 hours.

### Cell lines

In addition, this study conducted scratch assay, Transwell migration assay, and MTT proliferation cell assay. The widely recognized human MCF-7 breast cancer cell line was purchased from ATCC and cultured in the Roseville Park Memorial Institute 1640 (RPMI-1640) medium containing 10% -20% fetal bovine serum (FBS). The constant temperature of 37 °C was maintained in a 5% CO_2_ environment.

### Lentiviral Vector-Mediated ADSS Knockdown

Nanning Genesis Biotechnology Co., Ltd. has constructed small interfering RNA (siRNA) encoding targeted human ADSS and lentiviral expression plasmids encoding green fluorescent protein (GFP). Two types of siRNA ADSS plasmids hADSS-1304-s were constructed: AGCUCAAAUUCCAGUUAA (dT) (dT) and HADSS-1304-a: UUAACUGAAUUUGAAGCU (dT) (dT). As a negative control, a lentivirus expression plasmid encoding only GFP (GFP lentivirus) was also prepared. After 48 hours, the knockout efficiency of cells infected with ADSS siRNA encoding virus was confirmed using quantitative real-time polymerase chain reaction (qRT PCR) and Western blotting.

### Lentivirus-Mediated ADSS Overexpression

Engineering the expression plasmid of FLAG labeled ADSS using pMSCV IRES-GFP vector (Hanyin), and transfecting the plasmid or corresponding empty vector as NC into MCF-7 cells. Recombinant retroviruses from these cells were used to infect MCF-7 cells at a fold of 1, resulting in ADSS overexpressing (ADSS-OE) cells or NC cells.

### Quantitative Polymerase Chain Reaction (qPCR)

The tissue was ground in liquid nitrogen and homogenized with TRIzol; cells were lysed directly in culture plates with TRIzol; the homogenized samples were allowed to stand at room temperature for 10 minutes to separate nucleic acid-protein complexes; chloroform was added, and samples were centrifuged after shaking to separate them; the upper aqueous phase was taken and isopropanol was added to precipitate RNA; RNA precipitate was washed with 75% ethanol, dried, and then dissolved in RNase-free water or 0.5% SDS; RNA concentration and purity were determined using a micro-nucleic acid detector. MonScript™ 5×RTIII All-in-One Mix, template RNA, MonScript™ dsDNase, Nuclease-Free Water; incubation at 37 °C for 2 minutes to remove genomic DNA contamination; incubation at 55 °C for 15 minutes for cDNA synthesis; incubation at 85 °C for 5 minutes to deactivate DNA polymerase. Prepare the qPCR reaction system: TB Green® Premix Ex Taq™ II, Primer F, Primer R, cDNA template, RNase-Free ddH_2_O; calculate the primer annealing temperature; pre-treatment at 95 °C for 30 seconds, PCR cycles (40 cycles): 95 °C for 10 seconds, annealing at 60 °C for 20 seconds, extension at 72 °C for 30 seconds; perform melt curve analysis; record the Ct value of each sample and calculate the 2-ΔΔct value.

### Western blot

Remove the culture medium, wash with PBS, lyse the cells, centrifuge the lysate, and collect the supernatant; prepare the lysis solution, homogenize tissue samples, centrifuge after lysis, and collect the supernatant for protein quantification; run electrophoresis at 80V constant pressure, allow the bromophenol blue to migrate to the separating gel, increase the voltage to 100-120V, and run until the bromophenol blue runs out; block the membrane with blocking solution for 1 hour; dilute the primary antibody and incubate overnight at 4°C; incubate with an HRP-conjugated secondary antibody at room temperature for 1-2 hours; mix reagents A and B, and place the membrane protein-side down in contact with the mixture; place the membrane in the imaging system dark box for imaging and analysis.

### Immunohistochemistry

Place the slices baked overnight at 62 °C on the slicing rack, and sequentially place them in dewaxing solution I, dewaxing solution II, and dewaxing solution III for 10 minutes each. Then soak them in gradient ethanol (100%, 95%, 85%, 75%) once for 5 minutes each time, rinse with running water for 2 minutes, and soak them in double distilled water for 5 minutes. Dilute 20 x EDTA antigen retrieval solution with double distilled water to 1 x as needed, adjust the pH to 9.0, take an appropriate amount of working solution and preheat it to 95-98 °C in a microwave oven in the antigen retrieval kit. Place the tissue slices in preheated 1 × antigen retrieval solution, place the antigen retrieval kit in a microwave oven and heat it on low heat for 5 minutes, then continue heating for 10 minutes. After completion, cool naturally to room temperature. Wash with PBS for 3 × 5 minutes. At room temperature, the slices were incubated with 3% H_2_O_2_ (prepared with double distilled water) in the dark for 10 minutes.

Wash with PBS for 3 × 5 minutes. Use a tissue marker to draw a small circle along the outer contour of the tissue, with a distance of 2-3 mm from the tissue, to prevent the diffusion of subsequent incubation liquid and ensure sufficient incubation. Add 5% goat serum (diluted with 1 x PBS) dropwise to coat the sample, place the slices in a wet box, and incubate at 37 °C for 1 hour. Incubate the primary antibody with a blocking solution (5% goat serum) and dilute it to an appropriate concentration. The volume of reagents used in the specific experiment can be adjusted according to the size of the tissue sample. Pour the sealing solution onto the surface of the slice without washing. Drop 100 μ L of primary antibody diluent onto the surface of the glass slide to cover the tissue. Place the slice in a wet box and incubate overnight at 4 °C. On the second day, PBS was washed at room temperature on a horizontal shaker for 3 × 5 minutes; Prepare the secondary antibody diluent using the same method. Drop 100 μL of the secondary antibody working solution onto the surface of a glass slide to cover the tissue. Place the slices in a wet box and incubate at 37 °C for 30 minutes. After incubation, wash the PBS slides at room temperature on a horizontal shaker for 3 × 5 minutes. Add 100 μL of freshly prepared DAB color solution dropwise to the slices (mix the DAB substrate solution and concentrated DAB solution in a ratio of 1:20, prepare and use immediately). Control the color development time under the microscope. When the target protein gradually develops color but the background does not, rinse the slices with tap water to terminate the color development. Slices were stained with hematoxylin staining solution for about 1 minute, immediately washed with pure water, differentiated with hematoxylin differentiation solution for 2 seconds, immediately washed with pure water, and then returned to blue with hematoxylin blue solution for a few seconds, washed with pure water. Dehydrate the slices in 75%, 85%, 95%, anhydrous ethanol I, anhydrous ethanol II, and anhydrous ethanol III for 5 minutes each, and clear liquid I, transparent liquid II, and transparent liquid III for 5 minutes each. Cover with neutral gum and let it air dry naturally. Microscopic examination under an optical microscope. Staining result judgment: Under the microscope, the cell nucleus appears blue, while the positive result shows varying shades of brown. Immunohistochemistry images were analyzed using Image Pro plus 6.0 software, and data analysis was performed using SPSS 25.0 software. GraphPad Prism 11.0 was used for presentation. The data results are presented as mean and standard deviation. Independent t-test was used to compare the differences between the two groups, with *p*<0.05 indicating statistical significance.

### MTT (3-(4,5-Dimethylthiazol-2-yl)-2,5-diphenyltetrazolium bromide)

Rinse the cells twice with PBS; add 1mL of trypsin to cover the cell surface, and place in the incubator for 2 minutes; after cell shrinkage, rounding, and detachment, add 2mL of complete culture medium to stop digestion, and disperse the cells; collect the dispersed cells into a 15mL centrifuge tube after obtaining a single-cell suspension, centrifuge at 1000 rpm for 5 minutes; resuspend in PBS, repeat twice; finally, resuspend the cells in complete culture medium and set aside; count the cells using a cell counter: adjust the cell density to 5×10³ cells/well, and plate; continue culturing in a 37°C, 5% CO_2_ incubator for 16 hours; add the test drug at an appropriate concentration and continue incubating for 1 hour; remove the supernatant, add 90 μL of fresh culture medium, then add 10 μL of MTT solution, and continue incubating for 4 hours; remove the supernatant, add 110 μL of Formazan solubilization solution to each well, shake at low speed on a shaker for 10-15 minutes until the crystals are completely dissolved; measure the absorbance at 490 nm using an enzyme-linked immunosorbent assay reader for each well; calculate cell viability = [(OD of experimental well - OD of blank well) / (OD of control well - OD of blank well)] × 100%.

### Transwell

Retrieve the cell cryovial from the liquid nitrogen tank, place it in a 37°C water bath until the crystals completely disappear, spray with alcohol, and place in a clean laminar flow hood; transfer the cells into a 15 mL centrifuge tube containing 3 mL of complete culture medium, centrifuge at 1000 rpm for 5 minutes; discard the supernatant, resuspend the pellet in 5 mL of complete culture medium, transfer to a T25 culture flask, and place in a 37°C, 5% CO_2_ incubator for cultivation; discard the culture medium in the T25 flask; rinse the T25 flask once with PBS; add 5 mL of complete culture medium; mix Matrigel matrix gel with DMEM medium at a 1:8 ratio; evenly add 100 µL of the mixture onto the wells, and incubate in the incubator for 30 minutes; when the cell density in the T25 flask reaches 70%-80%, start preparing the cell suspension; discard the culture medium, rinse twice with PBS; add 1 mL of trypsin-EDTA (0.25%), and let it stand for 3 minutes.

Add 2 mL of complete culture medium to stop digestion, and disperse; collect the cells in a 15 mL centrifuge tube, and centrifuge at 1000 rpm for 5 minutes; resuspend in PBS twice; discard the supernatant, resuspend in serum-free culture medium; count using a cell counter, adjust to 2×10^5 cells/mL; add 500 µL of complete culture medium to a 24-well plate, place the wells in the plate ensuring no air bubbles, add 200 µL of cell suspension into each well, and incubate in a 37°C, 5% CO_2_ incubator for 48 hours; remove the wells, aspirate the upper medium, wipe the cells inside the wells with a cotton swab, wash the plate once with PBS, add 600 μL of 4% paraformaldehyde for fixation for 30 minutes; aspirate the fixative, air dry, add 500 μL of 0.1% crystal violet for staining for 30 minutes, remove the wells, rinse three times with PBS, wipe the cells on the bottom surface of the wells, allow the wells to dry, randomly select 3 fields for photography.

### Scratch assay

Retrieve the cell cryovial from the liquid nitrogen tank, place it in a 37°C water bath until the crystals completely disappear, spray with alcohol, and place in a clean laminar flow hood; transfer the cells into a 15 mL centrifuge tube containing 3 mL of complete culture medium, centrifuge at 1000 rpm for 5 minutes; discard the supernatant, resuspend the pellet in 5 mL of complete culture medium, transfer to a T25 culture flask and incubate in a 37°C, 5% CO_2_ incubator; discard the culture medium in the T25 flask, rinse once with PBS, then add 5 mL of complete culture medium; when the cell density in the T25 flask reaches 70%-80%, begin the plating process; draw three parallel horizontal lines at the bottom of each well in a six-well plate; discard the culture medium in the T25 flask, rinse twice with PBS, add 1 mL of trypsin-EDTA (0.25%), shake gently, let stand for 3 minutes, then add 2 mL of complete culture medium to stop digestion, disperse the cells, collect them in a 15 mL centrifuge tube, centrifuge at 1000 rpm for 5 minutes, discard the supernatant, resuspend in complete culture medium, count using a cell counter, adjust to 1×10^5 cells/mL; add 1 mL of complete culture medium to each well in the six-well plate to moisten the plate, then add 1 mL of cell suspension; once the cell density in the six-well plate reaches 100% on the following day, use a 200 µL pipette tip to draw lines perpendicular to the cell plane and the reference line on the back of the plate, rinse the cells 3 times with PBS to remove cells from the lines, add serum-free culture medium; take photographs under a 4x magnification, ensuring the lines are centered and vertical, maintain consistent background, and take samples at 0, 12, 24, 48 hours for photography.

### Data resources and preprocessing

Transcriptional data related to BRCA were obtained from The Cancer Genome Atlas (https://portal.gdc.cancer.gov/) 11 and included a total of 1104 BRCA tissue samples (from the BRCA group) and 113 paracancerous tissues (from the control group). In addition, to reveal molecular differences among subtypes, we performed a comparative analysis of gene expression across multiple BRCA subtypes by downloading the dataset GSE231629, including normal breast tissue, Luminal A, Luminal B, HER2 positivity, and TNBC. For data normalization and preprocessing, we used the normalizeBetweenArrays function of the limma software package 12.

### Receiver operating characteristic (ROC) analysis

The ROC curve is a useful tool for evaluating classifiers in biomedical and bioinformatics applications. Using the r package pROC 13, we used an analysis to evaluate the diagnostic performance of potential biomarkers. The results were plotted as ROC curves. In this study, we used this approach to assess the potential of ADSS as a diagnostic biomarker for BRCA. When the area under the curve (AUC) > 0.5, a higher AUC value closer to 1 indicates better diagnostic performance.

### Survival curve analysis

In this study, we conducted overall survival (OS) and recurrence-free survival (RFS) analyses of BRCA patients using the survminer and survival packages, and to visualize the results as survival curves.

### Differential gene expression analysis of genes and microRNAs (miRNAs)

To explore the BRCA dysregulation genes associated with high ADSS expression, we used the median ADSS expression value as a reference. ADSS expression values higher than the median were defined as high ADSS expression, while values lower than the median were defined as low ADSS expression; this median distinguishes BRCA samples into those with high-low ADSS expression. Differential expression analysis of miRNAs and RNAs in BRCA-Control and ADSS high-low expression samples was performed using the limma software package.

### Weighted gene coexpression network analysis

To explore the relationship between ADSS and BRCA, we used the WGCNA algorithm from the R package 14 to construct a coexpression network based on the ADSS. Through cluster analysis, we created a scale-free network. Module feature genes were used to estimate the correlation between modules and clinical features. We selected significant modules based on a *p* value < 0.01 and each gene in the module.

### Gene Ontology and Kyoto Encyclopedia of Genes and Genomes pathway enrichment

In this study, we conducted Gene Ontology (GO) and Kyoto Encyclopedia of Genes and Genomes (KEGG) pathway enrichment analyses of the module genes of interest. This analysis was developed using the clusterProfiler package in R 15. Functional terms or pathways with a *p* value < 0.05 were considered significantly enriched.

### Gene set enrichment analysis

Gene set enrichment analysis can assess the distribution trend of genes in a gene expression matrix based on their correlation with predefined gene sets and phenotypes, thus determining their contribution to the phenotype 16. We used the c5.bp.v7.0.entrez.gmt and c2.cp.kegg.v7.0.symbols.gmt data from the Molecular Signatures Database (MSigDB) (https://www.gsea-msigdb.org/gsea/msigdb/index.jsp) 17 as reference genomes and input phenotypes and whole-genome expression profiles for gene set enrichment analysis to validate the signaling pathways significantly activated or inhibited in BRCA.

### Cox univariate and multivariate analysis

Before Cox analysis, we performed a survival analysis of the module genes of interest. We selected 50 genes associated with prognosis and a total of 51 ADSS related genes, defined as scoring genes. Subsequently, we used a univariate Cox model to regress these scoring genes and employed multivariate Cox stepwise regression analysis to obtain ADSS-based scoring genes with a statistically significant impact on prognosis (*p*<0.05). In this study, we first identified features significantly associated with prognosis via Cox univariate analysis, identified independent prognostic factors via Cox multivariate analysis, and constructed an ADSS-based scoring model.

### Constructing a clinical indicator model using a nomograms to predict disease risk

Based on univariate and multivariate analyses, we constructed column line graphs using multiple clinical indicators and the ADSS index to predict the 3-, 5-, and 8-year survival rates of BRCA patients. These column line graphs were constructed using the "rms" and "survival" R packages 18. Survival was then evaluated using the Kaplan‒Meier survival curve 19 and the log-rank test.

### Identification of upstream regulators

In this study, the upstream regulators associated with the genes according to the ADSS score were explored via the hypergeometric test method using the RNAInter, TRRUST, STRING, and DrugBank databases and other databases as background sequences.

### Relationship between ADSS expression and immune infiltration in BRCA

Based on CIBERSORT (https://cibersort.stanford.edu/), immune cell infiltration estimation analysis was conducted to analyze the abundance of immune cells in BRCA samples.

### Construction of the multiomic regulatory landscape

To elucidate the single nucleotide polymorphisms of ADSS-based scoring genes in BRCA, the R package maftools 20 was used to visualize the single nucleotide polymorphisms and mutation details of these genes, as well as to display the copy number variation of these genes in BRCA patients. Additionally, correlation analysis was performed to explore the correlation between the methylation level and transcription level of ADSS-based indicator genes.

### Immunohistochemical map analysis

In this study, an immunohistochemical map of BRCA was obtained based on information from the human protein atlas (https://www.proteinatlas.org/) 21.

### Data analysis and statistics

All bioinformatics analyses in this study were performed on the Bioinforcloud platform (http://www.bioinforcloud.org.cn).

## Results

### The prognostic effects of the ADSS in BRCA

**Figure 1:**
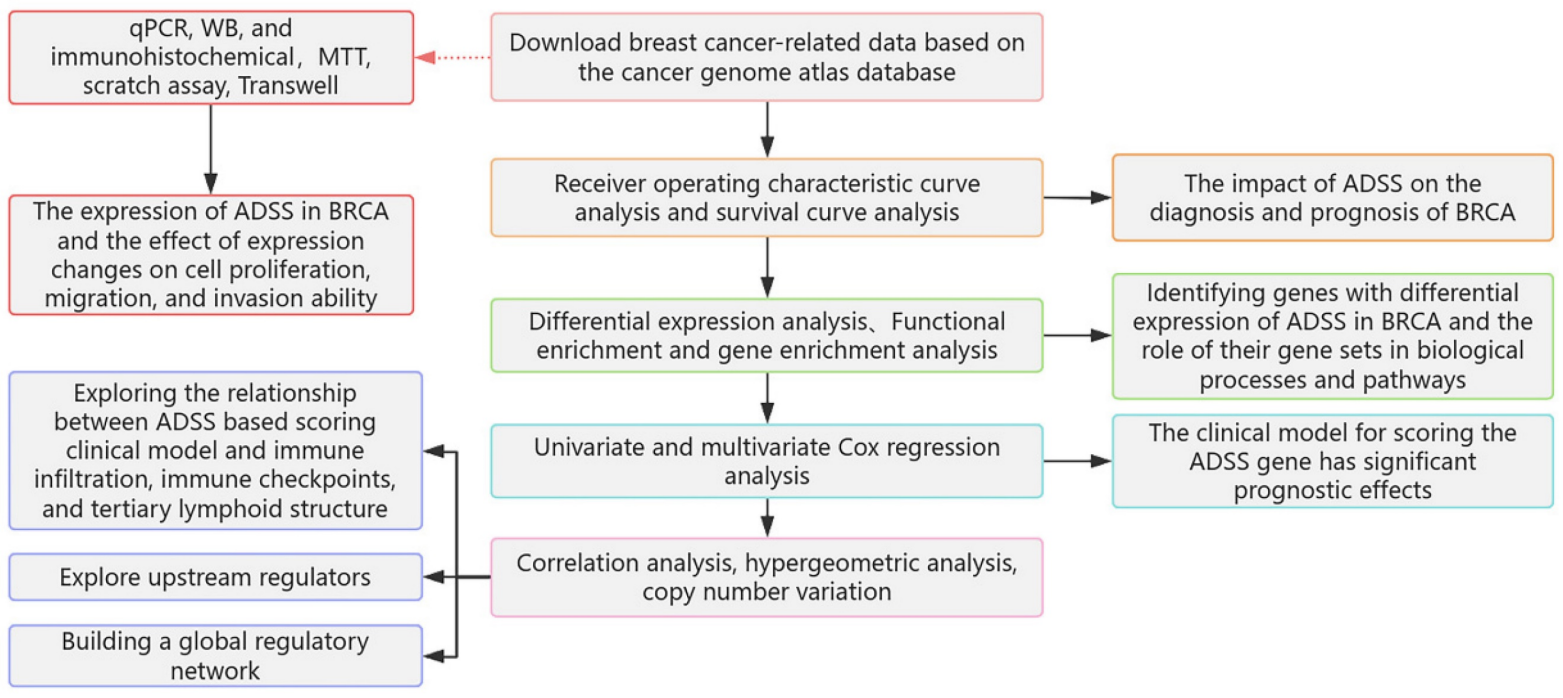
Flowchart.

**Figure 2:**
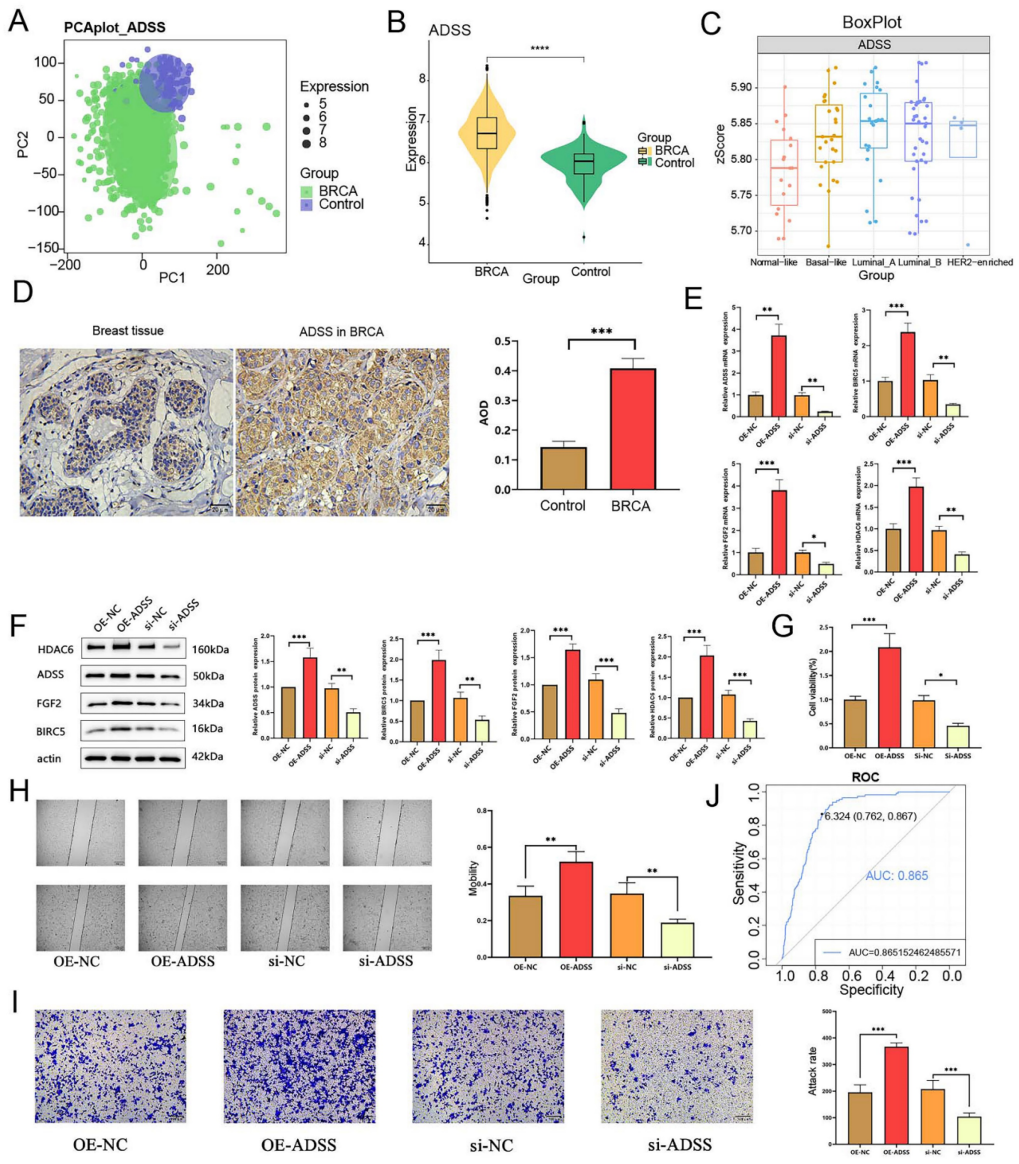
** The prognostic effects of adenylosuccinate synthetase (ADSS) in breast cancer (BRCA).** F2A. Principal component analysis density plot showing the expression values of ADSS. F2B. Violin plot showing the transcription levels of ADSS in BRCA. F2C. The violin plot shows the transcription levels of ADSS in BRCA subtypes. F2D. Immunohistochemical detection of ADSS expression trend in tissue samples. F2E. qPCR assay was used to detect the expression of proliferation, invasion, and metastasis related genes in MCF-7 cells overexpressing/knocking down ADSS. F2F. WB assay was used to detect the expression of proliferation, invasion, and metastasis related genes in MCF-7 cells overexpressing/knocking down ADSS. F2G. MTT assay for detecting the effect of ADSS overexpression/knockdown on cell proliferation in MCF-7 cells. F2H. Scratch assay explores the changes in migration of MCF-7 cells overexpressing/knocking down ADSS. F2I. Transwell experiment explores the changes in migration of MCF-7 cells overexpressing/knocking down ADSS. F2J. Diagnostic receiver operating characteristic curve illustrating the diagnostic potential of the ADSS for BRCA. (*Represents *p*<0.05, * * represents *p*<0.01, * * * represents *p*<0.001).

The flow of this study is shown in Figure 1. Initially, our research indicates that there is significant differentiation between the BRCA group and its subgroups compared to the control group (Figure 2A). The expression level of ADSS is significantly elevated in BRCA and its subtypes (Figures B-C), suggesting that the expression pattern of ADSS may be the same across BRCA and its subtypes. Immunohistochemistry experiments detected higher ADSS staining levels in BRCA (Figure 2D), indicating higher ADSS protein levels in BRCA. Furthermore, qPCR and Western blot analyses revealed that overexpression of ADSS significantly increased the expression levels of proliferation-related gene BIRC5 and invasion-metastasis related genes FGF2 and HDAC6 (Figures 2E-F). MTT assay results showed that high expression of ADSS can promote the proliferation of BRCA cells (Figure 2G). Transwell and scratch tests revealed the significant enhancement of ADSS on the invasive and migratory capabilities of BRCA cells (Figures 2H-I). Finally, the AUC of the model reached 0.865, indicating that ADSS has a good diagnostic effect on BRCA (Figure 2J), suggesting that ADSS may be a potential diagnostic marker for the disease. In summary, our research results emphasize the important role of ADSS in BRCA and its subtypes and provide new molecular markers for future clinical diagnosis and treatment.

### The regulatory role of the coexpression module of the ADSS gene in BRCA

**Figure 3:**
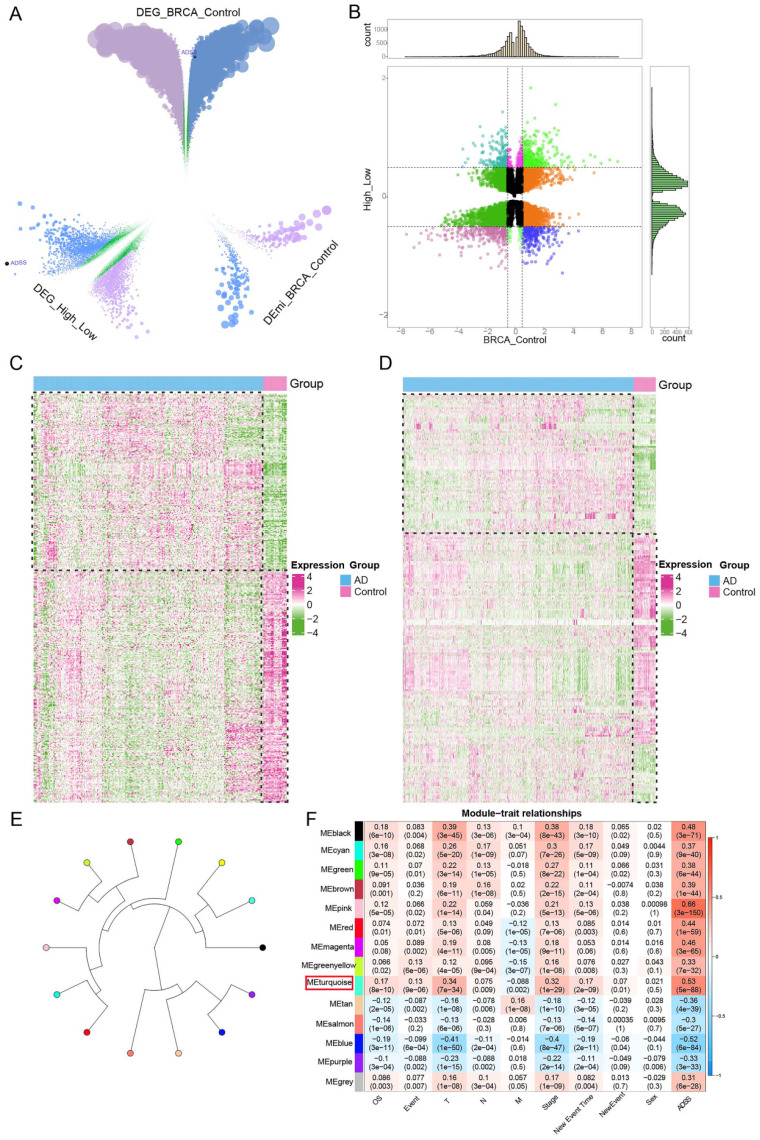
** Regulatory role of ADSS coexpression modules in BRCA.** F3A. Volcano plot displaying the differentially expressed genes and differentially expressed miRNAs in the control and BRCA patients, where green represents nonsignificant genes, purple represents upregulated genes, and blue represents downregulated genes. F3B. Nine-panel scatter plot illustrating the potential interaction of ADSS with dysregulated genes and miRNAs in BRCA. F3C. Heatmap displaying the expression patterns of positively and negatively regulated genes in the control group and high-low ADSS expression group. F3D. Heatmap displaying the expression patterns of positively and negatively regulated miRNAs in the control group and high-low ADSS expression group. F3E. Circular tree plot of module adjacency showing the interaction between ADSS and coexpressed modules of regulatory genes in BRCA. F3F. Heatmap of module correlation revealing the correlation of gene coexpression modules with ADSS, OS, Stage, T, N, M, Event and sex.

We identified a total of 28,952 differentially expressed genes (DEGs) and 695 differentially expressed miRNAs (DEmiRNAs) in the samples (Figure 3A). To further explore the impact of high ADSS expression on BRCA, we also identified DEGs and miRNAs between samples with high ADSS expression and those with low ADSS expression, which resulted in 9,915 DEGs and 3 DEmiRNAs. Subsequently, 3,435 DEGs were consistently upregulated or downregulated, and 3 DEmiRNAs were consistently upregulated or downregulated in both sets of differential expression data; these were defined as the genes whose expression was dysregulated in BRCA patients and the DEmiRNAs associated with high expression of ADSS (Figure 3B). A heatmap was constructed to show that high expression levels of BRCA-dysregulated genes and miRNAs were associated with high expression of ADSS (Figure 3C-D).

On the basis of WGCNA, we used BRCA related ADSS DEGs (*p*<0.05) to cluster dysregulated genes affected by high ADSS expression into 14 coexpression modules (Figure 3E), obtained the coexpression module genes most strongly associated with ADSS, and further calculated the correlation of each color module with the ADSS and clinical characteristics. The comprehensive module and ADSS, tumor primary foci, stage, and other clinical characteristics were significantly positively correlated with the turquoise module and the ADSS (r=0.53, p=5e-88) (Figure 3F); therefore, the turquoise module may be a key module of ADSS-mediated poor prognosis, and further research is needed.

### Biological functions and signaling pathways regulated by the ADSS in BRCA

**Figure 4:**
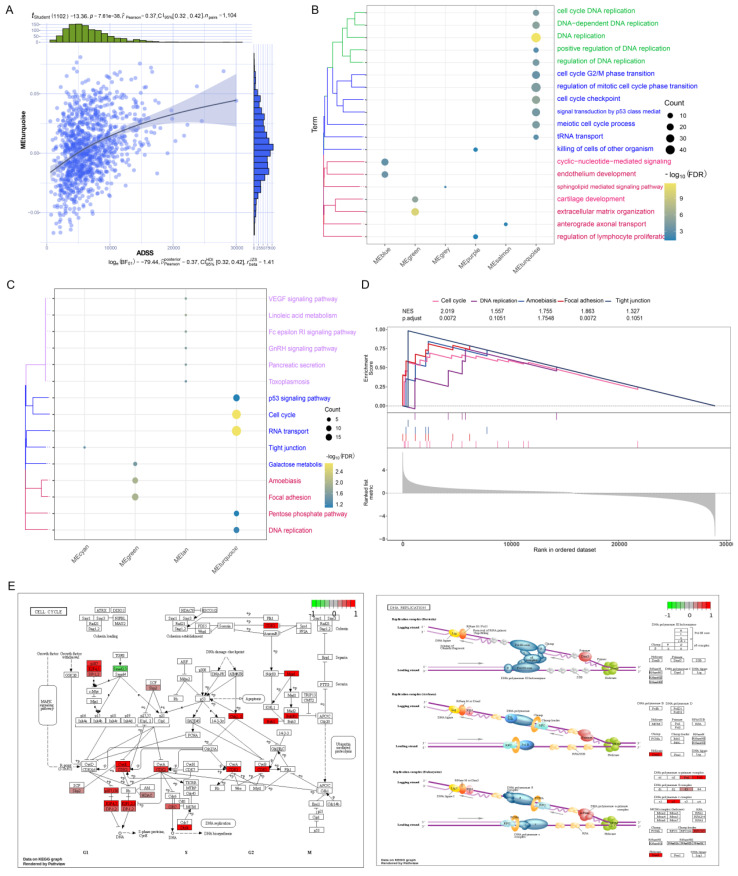
** Biological functions and signaling pathways regulated by ADSS in BRCA.** F4A. A series correlation scatter plot illustrating the correlation with coexpressed modules of the ADSS. F4B. Clustered bubble plots demonstrating the biological progression of ADSSs involved in different coexpression modules. F4C. The cluster bubble plots demonstrate that the Kyoto Encyclopedia of Genes and Genomes (KEGG) signaling pathways significantly activated by ADSS in different coexpression modules. F4D. Composite gene set enrichment analysis plots demonstrating that the KEGG signaling pathway significantly activated by ADSS in different coexpression modules. F4E. Pathway map plots showing KEGG signaling pathways significantly activated by ADSS in different coexpression modules.

The turquoise module was significantly positively correlated with the ADSS score (Figure 4A). Therefore, we further explored the biological functions and signaling pathways of the genes related to the turquoise module. Through enrichment analysis, we found that the genes in the turquoise module were significantly enriched in biological processes related to DNA replication and the cell cycle (Figure 4B). We also found significant enrichment of the cell cycle, RNA transport, and p53 signaling pathways (Figure 4C). Among them, DNA replication, the cell cycle, and RNA transport are associated with cancer metastasis and occurrence 22. To verify whether these pathways were significantly regulated, we further conducted gene set enrichment analysis and found that the cell cycle, DNA replication were significantly activated (Figure 4D). We also displayed these activated signaling pathways through pathway maps (Figure 4E), which indicated that the pathways associated with these genes in the turquoise module can mediate BRCA development and may play an important role in this disease.

### Prognostic effect of the ADSS score clinical model

**Figure 5:**
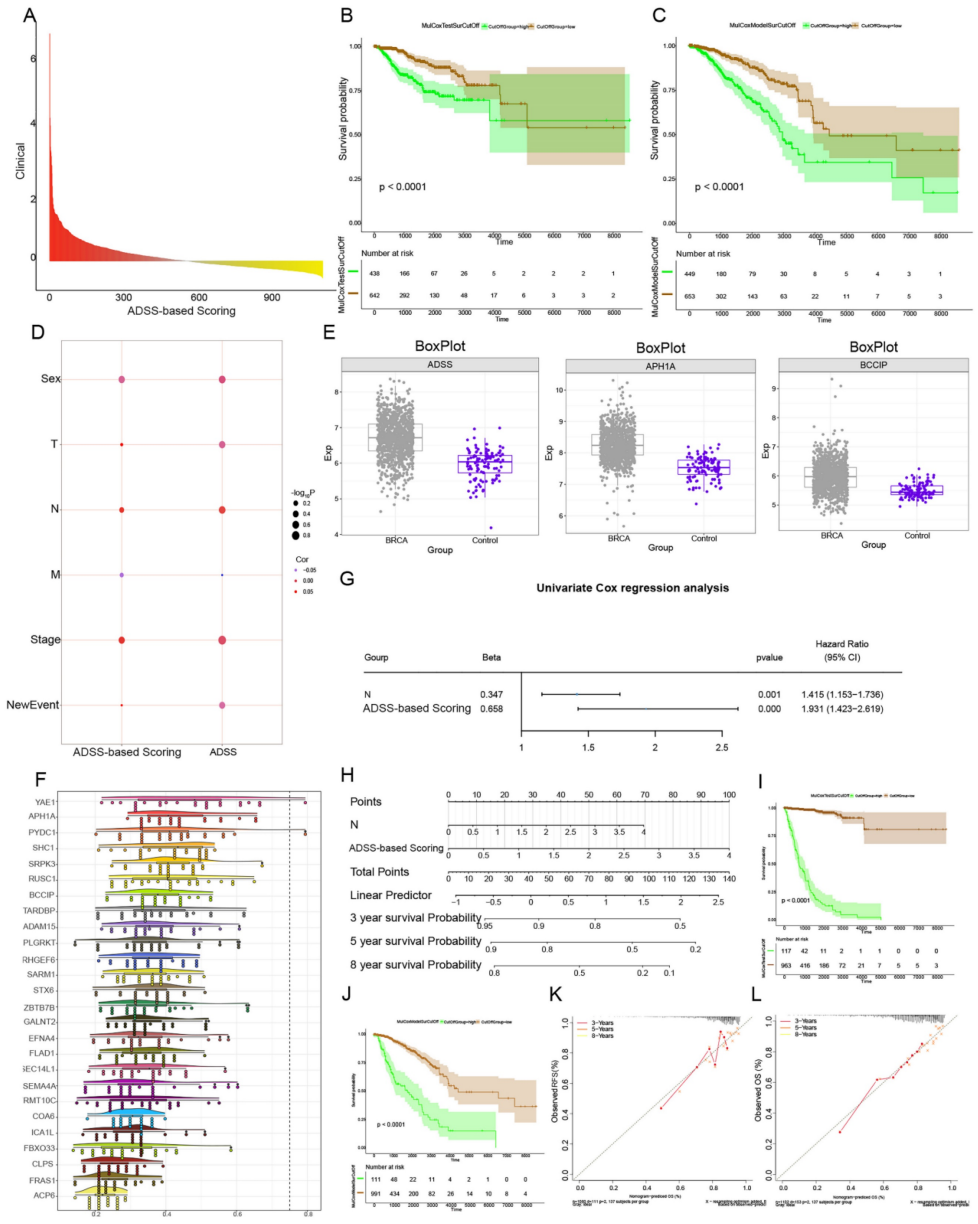
** Prognostic effects of the ADSS according to the clinical model.** F5A. Bar chart showing the Cox multivariate prognostic score of the genes coexpressed with the ADSS. F5B-C. Survival curve displaying the RFS (B) and OS (C) survival curves based on the ADSS-based scoring genes. F5D. Bubble plot showing the correlation between Cox prognostic score and clinical indicators. F5E. Box plot displaying the transcription levels of the genes. F5F. Cloud and rain plots showing the biological associations of the scoring genes. F5G. Forest plot displaying the univariate prognostic efficacy of the ADSS-based score and clinical indicators. F5H. Line plot displaying the clinical model of the ADSS-based score. F5I-J. Survival curve showing the prognostic potential of the clinical model for RFS (I) and OS (J) based on the ADSS-based score. F5K-L. Calibration curve showing the prognostic potential of the clinical model for RFS (K) and OS (L) based on the ADSS-based score.

Next, we performed RFS and OS survival curve analysis for patients with ADSS scores and significant module genes, screened the top 50 genes with prognostic significance, and then performed multivariate Cox regression analysis of the 51 genes with a score in the ADSS to obtain the score for the ADSS model. We further divided patients into high-low groups (p <0.001) (Figure 5A-C), which indicated that the higher the ADSS-based score was, the worse the prognosis was. The correlation analysis revealed that tumor primary lesion, metastatic lymph node (N), and stage were significantly positively correlated with the ADSS-based score in patients (Figure 5D). Subsequently, we calculated and evaluated the expression values of the genes in the different groups and observed higher transcription levels of the ADSS score genes in the BRCA group (Figure 5E). Several genes, such as YAE1, PYDC1 and SRPK3, had strong biological associations with ADSS (Figure 5F). We also found that N according to the ADSS-based score (*p* value=0.001, hazard ratio=1.415) was an independent risk factor for BRCA, and the ADSS-based score, like the N clinical index, could be used as an independent prognostic factor for BRCA (Figure 5G). N and ADSS-based scoring can predict the 3-, 5-, and 8-year survival of BRCA patients (Figure 5H). Survival curve analysis revealed that the ADSS clinical score model has the potential to predict BRCA prognosis (Figure 5I-J), while calibration curves confirmed the predictive accuracy of the column line chart (Figure 5K). In conclusion, these results indicate that the ADSS clinical score model has significant prognostic potential for BRCA patients.

### Abnormal expression of clinical module genes of the ADSS affects the BRCA immune microenvironment

**Figure 6:**
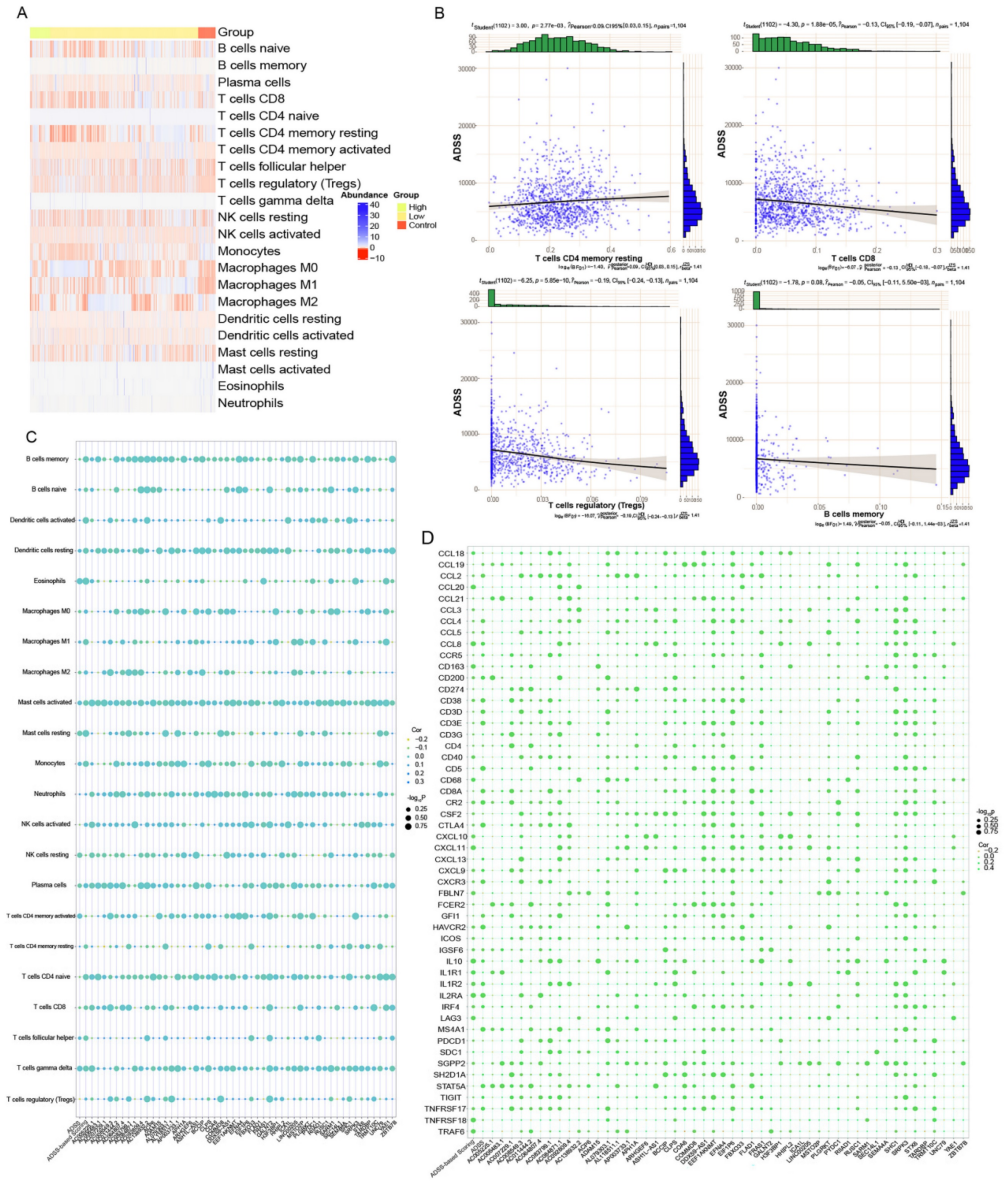
** Abnormal expression of the ADSS in the clinical module affects the immune microenvironment in breast cancer.** F6A. Heatmaps showing the infiltration abundance of immune cells in the control and high-low-ADSS-based clinical score groups. F6B. Scatter plots showing the correlation between immune cell infiltration abundance and ADSS expression. F6C. Bubble plots showing the correlations between ADSS-based score, score, immune checkpoint-related genes, and tertiary lymphoid structural markers. F6D. Bubble plot showing the correlations between ADSS-based score, score-related genes, and immune cell infiltration levels.

We performed immune cell infiltration analysis on control and the high-low-expression group ADSS-based clinical scoring genes and determined the infiltration abundance of immune cells (Figure 6A). Through correlation analysis, we found a negative correlation between ADSS score and immune cells, such as CD8^+^ T cells and B cells, suggesting that ADSS may inhibit the infiltration of some immune cells (Figure 6B). In addition, genes identified by ADSS-based clinical scoring were significantly associated with immune cell infiltration, immune checkpoint-related genes, and tertiary lymphoid structure genes (Figure 6C-D). In conclusion, a higher ADSS-based clinical score may be involved in the immune environment of patients, indicating that can be reprogrammed the immune environment by targeting ADSS or the scoring gene to inhibit BRCA progression.

### Upstream regulators of the clinical indicator model of the ADSS

**Figure 7:**
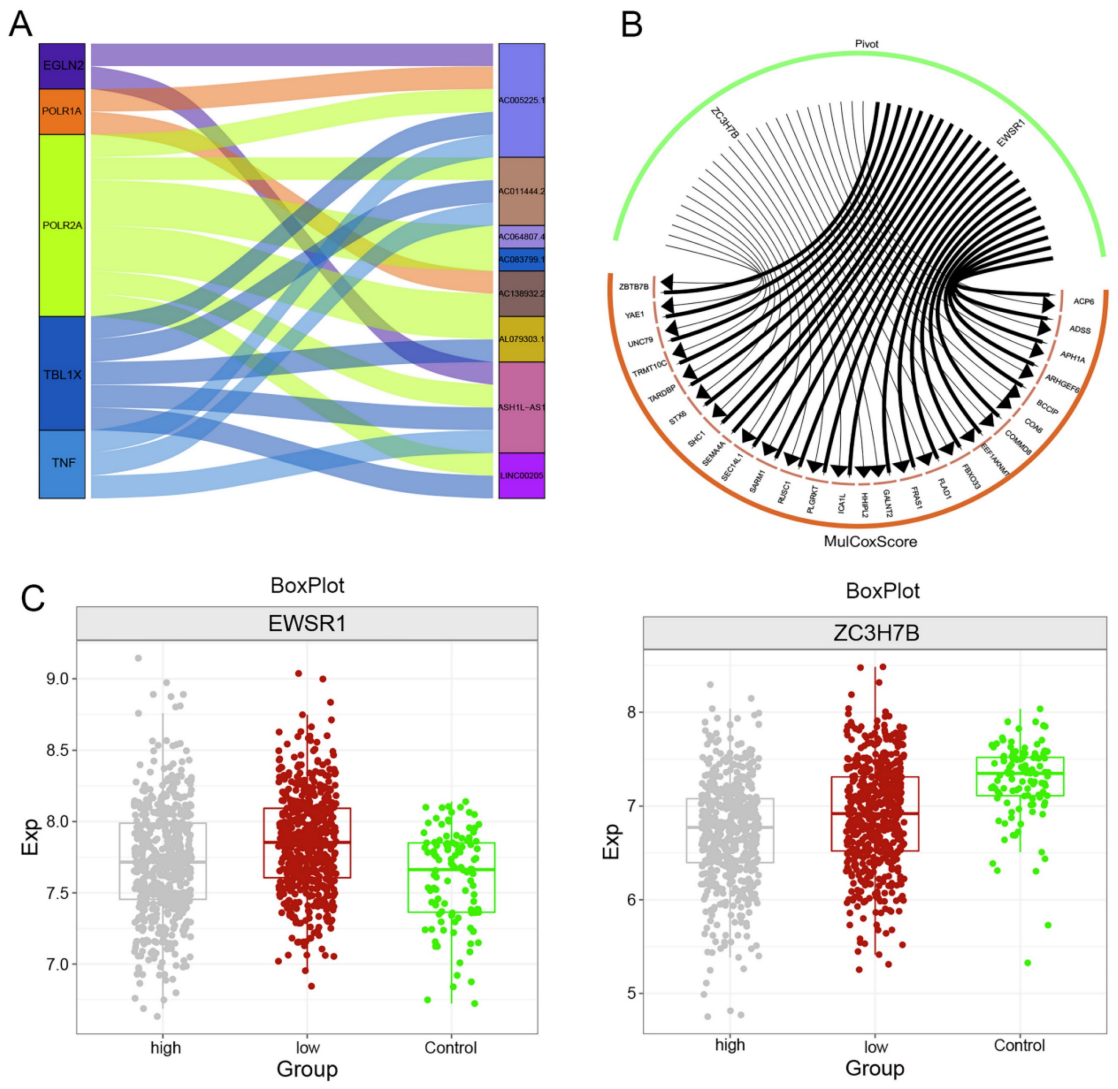
** Upstream regulators of clinical indicator genes for ADSS.** F7A. Sankey plot showing the regulatory effect of long noncoding RNAs on the expression of ADSS-based clinical scoring genes. F7B. A circular network plot shows the regulatory effect of RNA-binding proteins on the ADSS-based clinical scoring genes. F7C. Box plot - immunohistochemistry image showing the transcription levels of RNA-binding proteins.

We used the hypergeometric test method to explore the upstream regulatory factors of ADSS-based clinical scoring genes, including long noncoding RNAs (lncRNAs) and RNA binding proteins (RBPs). The Sankey diagram displays the upstream lncRNAs regulating the ADSS-based clinical scoring genes, including EGLN2, POLR1A, POLR2A, TBL1X, and TNF (Figure 7A). The RBPs included EWSR1 and ZC3H7B (Figure 7B); moreover, the EWSR1 expression level was greater in the group with low ADSS-based clinical scores, and the ZC3H7B expression level was greater in the control group (Figure 7C-D).

### Clinical indicator model of the multiomics global regulatory network of the ADSS

**Figure 8:**
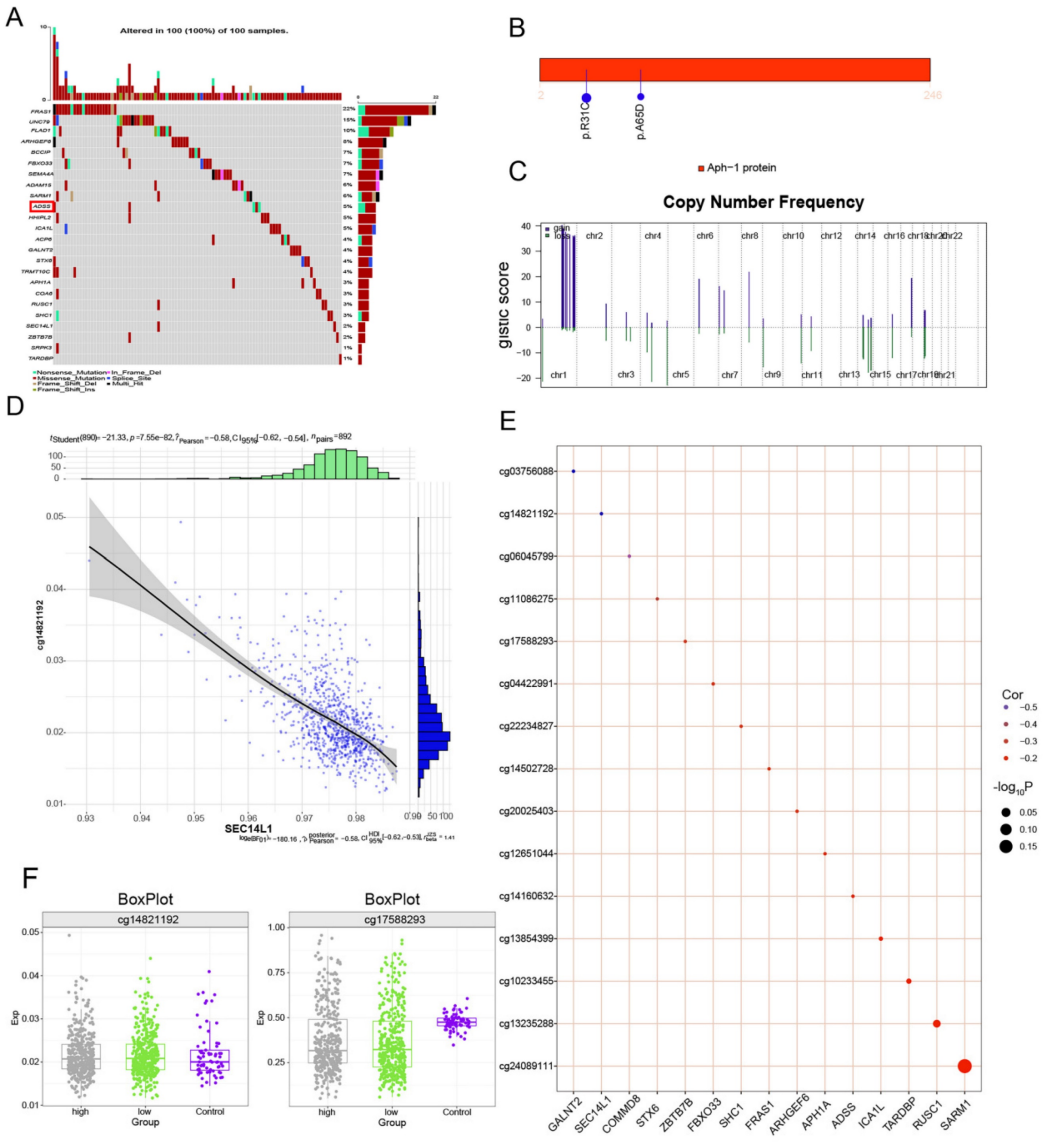
** Multiomics global regulatory network of clinical indicator genes for the ADSS.** F8A. Waterfall plot showing the mutational landscape of the ADSS-based clinical score gene global regulatory network in BRCA. F8B. Lollipop plot showing the detailed mutation information of the ADSS-based clinical scoring genes in BRCA. F8C. The chromosome bar plot shows the copy number spectrum of the ADSS-based clinical score gene global regulatory network in BRCA. F8D. Scatter plot showing the correlation between the methylation level and transcription level of the ADSS-based clinical score gene global regulatory network in BRCA. F8E. Bubble plot showing the correlation between methylation influence and ADSS-based clinical score. F8F. Box plot showing the methylation level of the ADSS-based clinical score gene global regulatory network in BRCA.

We continued to explore somatic mutations in ADSS-based clinical scoring genes and found that the mutation frequency in the ADSS cohort was 5% (Figure 8A); moreover, the mutation sites in the ADSS-based clinical scoring genes are shown in the lollipop chart (Figure 8B). In addition, ADSS-based clinical scoring genes have copy number increases and deletions in the global regulatory network (Figure 8C). We also found that ADSS-based clinical scoring genes were negatively correlated with the methylation sites cg03756088, cg14821192 and cg06045799 (Figure 8D-E). The methylation sites were highly expressed in the high-low group of ADSS-based clinical scoring genes and in the contrast group (Figure 8F).

## Discussion

BRCA is the most common malignant tumor in the world 4. BRCA is the disease with the highest cancer morbidity and mortality among women worldwide 23. In particular, the treatment options for estrogen receptor-negative BRCA and triple-negative BRCA are very limited, resulting in low survival rates 24. Therefore, research on BRCA-related genes can help reveal new and promising prognostic biomarkers and drug targets to improve the clinical prognosis of patients with BRCA 25. The purpose of this study was to identify the relevant module genes associated with ADSS in BRCA and use them to establish a clinical indicator model for the ADSS, further investigating the potential role of this model.

First, we found that the expression level of ADSS was high in BRCA tissues via immunohistochemical analysis, and further analysis of BRCA tissues and subtypes revealed that ADSS was highly expressed in BRCA and its subtypes; therefore, we speculated that this study is also applicable to BRCA subtypes. Second, the genes with ADSS associated in the turquoise module were significantly enriched in biological processes and pathways, especially in DNA replication and the cell cycle. DNA replication is essential for cell proliferation 26, and several studies have shown a significant correlation between DNA replication and BRCA 27. The process of cell division plays a crucial role in the development of cancer 28 and involves numerous regulatory proteins that guide cells through a series of specific events, ultimately leading to mitosis and the production of two daughter cells. The cell cycle can be altered by many viral agents, including polyomaviruses, papillomaviruses, and adenoviruses. Due to changes in oncogenes that indirectly affect the cell cycle or tumor suppressor genes or oncogenes that directly affect cell cycle regulation, the cell cycle is often poorly controlled during tumorigenesis 22. Because the turquoise module was significantly positively correlated with the ADSS score, primary tumor focus T stage and other clinical characteristics, we selected the turquoise module. Module genes that were significantly related to both RFS and OS were screened and subjected to enrichment analysis. We found that the ADSS in the turquoise module were significantly enriched in signaling pathways such as the p53 signaling pathway, the cell cycle, and RNA transport, which have been demonstrated to be involved in cell growth, proliferation, cancer invasion, metastasis, and poor patient survival 29. Multiple studies have shown that genes can affect cancer cell proliferation, apoptosis, and invasion by mediating these signaling pathways 30. Therefore, the ADSS may influence the proliferation, migration, and invasion of BRCA cells through signaling pathways such as the p53 signaling pathway, cell cycle, and RNA transport.

We also performed an infiltration analysis of immune cells according to the ADSS-based clinical score genes and demonstrated the correlation of immune cell infiltration with ADSS-based clinical score gene expression with a bubble map. The ADSS inhibits the infiltration of certain immune cells, such as CD8^+^ T cells and B cells, among which CD8^+^ T cells enhance antitumor immunity 31, 32 and have significant antitumor effects, inhibiting tumor growth and prolonging survival 33. These findings suggested that inhibiting the expression of the ADSS could promote immune cell infiltration and potentially inhibit BRCA progression. We also showed negative correlations between the ADSS and immune cell infiltration, immune checkpoint-related genes, and tertiary lymphoid structure genes. Immune checkpoint molecules are defined as ligand-receptor pairs that exert inhibitory or stimulatory effects on immune responses 34. They are crucial for maintaining self-tolerance and regulating the duration and intensity of immune responses in different tissues to minimize tissue damage. In the present study, the ADSS exhibited a negative correlation with the expression of these immune checkpoint molecules, indicating that the ADSS inhibits immune checkpoint gene expression. In conclusion, the ADSS and its related genes can impact the immune microenvironment of BRCA patients, promoting the development of this disease.

Finally, we used a hypergeometric test method to explore the upstream regulatory factors of the clinical indicator genes of the ADSS, including lncRNAs and RBPs. These regulatory factors have been proven to be significantly associated with BRCA. Increasing evidence suggests that noncoding RNAs, including miRNAs and lncRNAs, play important roles in tumorigenesis 35. Aberrations in lncRNAs have been shown to have inhibitory or tumorigenic effects; regulate tumor proliferation, invasion, and migration through various mechanisms; and are associated with the clinical pathological features of tumor patients 36. For instance, EGLN2 is a prolyl hydroxylase that contributes to the occurrence of BRCA and was recently identified as a transcriptional coactivator that regulates mitochondrial function under normal hypoxic conditions through interactions with NRF1 and PGC1A 37, 38. Furthermore, the tumor microenvironment plays a critical role in disease progression and progression through mechanisms such as inflammation. TNF-α is an important proinflammatory factor in the tumor microenvironment of BRCA patients and is primarily secreted by stromal cells, tumor-associated macrophages and cancer cells themselves. TNF-α is involved in epithelial-mesenchymal transition and metastasis in BRCA cells 39. Additionally, RBPs serve as fundamental elements in the functional cycle of cells and are involved in gene expression, translation, transcriptional regulation, and extracellular migration 40. For example, the expression of ZC3H7B, an RBP, and EWSR1 mutations have been proven to significantly influence the migration and occurrence of breast tumors 41, 42.

In summary, we established a new clinical scoring model to predict the prognosis of BRCA patients, providing a new reference for clinical treatment by healthcare professionals. The clinical index model based on the ADSS may be a useful tool for guiding the personalized management of BRCA patients and promoting personalized management. However, these findings have limitations, and additional studies should be performed to verify these findings in larger samples to determine whether the clinical scoring model of ADSS is also applicable in the clinical treatment of other BRCA patients.

## Abbreviations

BRCA: Breast cancer
ADSS: Adenylosuccinate synthetase
WGCNA: weighted gene coexpression network analysis
ROC: receiver operating characteristic
AUC: area under the curve
OS: overall survival
RFS: recurrence-free survival
miRNAs: microRNAs
DEGs: differentially expressed genes
DEmiRNAs: differentially expressed miRNAs
N: metastatic lymph node
lncRNAs: long noncoding RNAs
RBPs: RNA-binding proteins
KEGG: Kyoto Encyclopedia of Genes and Genomes
